# CDX2 regulates interleukin‐33 gene expression in intestinal epithelial cells (LS174T)

**DOI:** 10.1002/2211-5463.13161

**Published:** 2021-05-02

**Authors:** Sylvester Larsen, Jakob Benedict Seidelin, Johanne Davidsen, Katja Dahlgaard, Claus Henrik Nielsen, Eric Paul Bennett, Ole Birger Pedersen, Mehmet Coskun, Jesper Thorvald Troelsen

**Affiliations:** ^1^ Department of Science and Environment Roskilde University Denmark; ^2^ Department of Clinical Immunology Næstved Hospital Denmark; ^3^ Department of Gastroenterology, Medical Section Herlev Hospital University of Copenhagen Herlev Denmark; ^4^ Department of Surgical Gastroenterology Zealand University Hospital Køge Denmark; ^5^ Institute for Inflammation Research Center for Rheumatology and Spine Diseases, Section 7521 Copenhagen University Hospital Rigshospitalet Denmark; ^6^ Department of Odontology Copenhagen Center for Glycomics Faculty of Health Sciences University of Copenhagen Denmark; ^7^ Department of Biology The Bioinformatics Centre Biotech Research and Innovation Centre (BRIC) University of Copenhagen Denmark

**Keywords:** *Caudal*‐related homeobox transcription factor 2, gene regulation, inflammatory bowel disease, interleukin‐33, intestinal regulation, transcriptional control

## Abstract

Dysregulation of interleukin‐33 (IL‐33) has been implicated in the pathogenesis of several autoimmune and inflammatory diseases, but few studies have examined transcriptional regulation of the *IL33* gene. In the intestines, gene regulation is controlled by a transcription factor network of which the intestinal‐specific transcription factor CDX2 is a key component. In this study, we investigated whether CDX2 regulates *IL33* mRNA expression. We examined *IL33* mRNA expression in primary colonic epithelial cells from healthy humans and epithelial cell lines, revealing high expression levels in primary colonic and LS174T cells. Combining genomics data (ChIP‐seq, RNA‐seq) and *IL33* promoter analyses in LS174T cells revealed intronic enhancer activity in the *IL33* gene that is dependent on CDX2 expression. Western blotting and qRT‐PCR confirmed that *IL33* expression is upregulated in a CDX2 concentration‐dependent manner, thereby providing the first evidence that CDX2 regulates the expression of *IL33*.

## Graphical abstract

We investigated whether the intestinal‐specific master transcription factor CDX2 regulates *IL33* mRNA expression in intestinal epithelial cells, revealing high expression levels in primary colonic and LS174T cells. Combining ChIP‐Seq, RNA‐Seq, *IL33* promoter analyses, western blotting, and qRT‐PCR revealed both a CDX2‐dependent enhancer and a CDX2 concentration‐dependent upregulation of *IL33* expression, providing the first evidence of CDX2 regulating *IL33* expression.

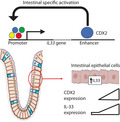

**Abbreviations**
CDX2: *Caudal*‐related homeobox transcription factor 2
HA: hemagglutinin
IBD: inflammatory bowel disease
IL‐33: interleukin‐33

Interleukin (IL)‐33 is a pleiotropic member of the IL‐1 family of cytokines and is constitutively expressed by a diverse range of cells including epithelial, endothelial, and fibroblast‐like cells [1]. It regulates metabolic homeostasis and intestinal inflammation through its ability to increase wound healing and goblet cell numbers and plays a role in inflammatory bowel disease (IBD) [1, 2]. IL‐33 is synthesized as a full‐length 30 kDa bioactive protein, but it can be cleaved or inactivated by caspases or proteases to modify its function [3]. Full‐length IL‐33 can be released to the extracellular environment by necrotic or damaged cells in response to injury, infections, or inflammation, where it acts as an ‘alarmin’ that amplifies immune responses [4]. However, intracellular full‐length IL‐33 may also act as a nuclear factor with transcriptional activity, as it was found that it binds to the p65 subunit of NF‐κB and reduces p65‐induced transcriptional activation [5].

Few studies have addressed the transcriptional regulation of the *IL33* gene. It has been shown that the IL‐33 promoter activity can be increased by interferon‐γ and that the IL‐33 protein expression can be stimulated by growth factors, transcription factors, and other proteins [6, 7]. In the intestines, gene regulation is controlled by a transcription factor network of which *Caudal*‐related homeobox transcription factor 2 (CDX2) is a key component. CDX2 is specifically expressed in the adult intestine, and it is essential in maintaining intestinal homeostasis to control the balance between cell proliferation and differentiation [8].

## Materials and methods

### Cultivation of cell lines and isolation of primary colonic epithelial cells

The human colon cancer cell lines Caco‐2, HT‐29, LS174T, SW480 and DLD‐1 (American Type Culture Collection, Rockville, MD, USA) and CDX2‐inducible LS174T cells [9] were cultured in Dulbecco's modified Eagle's medium supplemented with 10% FBS and 1% glutamine and maintained at 37 °C and 5% CO_2_. Primary colonic epithelial cells were isolated from four nonpregnant healthy people over the age of 18, by pooling six biopsies from either colon transversum or descendens taken at the same time during routine colonoscopy where all investigations subsequently turned out to be normal. The method was as described previously [10]. The biopsies were collected with informed written consent at the Department of Gastroenterology, Herlev Hospital, Denmark. The study methodologies conformed to the standards set by the Declaration of Helsinki and were approved by the Capital Region of Denmark Committee on Health Research Ethics.

### *CDX2* knockdown and induction with doxycycline

The CDX2‐inducible LS174T cells are described in Ref. [9]. In short, zinc finger nucleases were utilized to generate LS174T cells with a biallelic *CDX2* knockout. Subsequently, a cassette containing the *CDX2* gene under the control of a doxycycline‐activated Tet‐3G induction system was inserted into the AAVS1 safe‐harbor locus of one allele. Using this system, it is possible to control the expression of *CDX2* by stimulation with doxycycline without any leakiness [9].

### *IL33* qRT‐PCR and Luminex analysis

Total RNA was extracted (NucleoSpin RNA kit; Macherey‐Nagel, Düren, Germany) from Caco‐2, HT29, LS174T, CDX2‐inducible LS174T, SW480, DLD1, and isolated colonic epithelial cells. 200 ng RNA was used for cDNA with SuperScript III Reverse Transcriptase (Invitrogen, Paisley, UK). Quantitative RT‐PCR was performed using the Maxima SYBR Green qPCR Master Mix (Thermo Fisher Scientific, Waltham, MA, USA). All experiments were normalized to the housekeeping gene Ribosomal Protein Large P0 (RPLP0).

CDX2 expression in wild‐type and CDX2‐inducible LS174T cells was induced with 0, 2, 4, 6, 8, or 10 ng·mL^−1^ doxycycline for 24 h in quadruplicates. Growth media were stored at −80 °C before measuring IL‐33 protein using the Bio‐Plex Pro Human Th17 assay for IL‐33 (171‐BA012M).

### Construction of luciferase reporter constructs

The *IL33* promoter and enhancer sequences were PCR‐amplified and subsequently extended with In‐Fusion primers and gel‐purified (Table 1A). pGL4.10 vector (Promega, Madison, WI, USA) was digested with *Hin*dIII and gel‐purified. The pGL4.10‐*IL33*‐promoter plasmid was constructed by In‐Fusion cloning (Clontech, Fremont, CA, USA) using purified promoter DNA and digested vector in a 1 : 2 molar ratio. 2.5 µL cloning reaction was used for transformation of One Shot TOP10 chemically competent *Escherichia coli* (Thermo Fisher Scientific). DNA from colonies was isolated and Sanger‐sequenced (Beckman Coulter Genomics, High Wycombe, UK). For the construction of the pGL4.10‐*IL33*‐promoter+enhancer plasmid, the pGL4.10‐*IL33*‐promoter plasmid was digested with *Sal*I, gel‐purified, and cloned with enhancer insert in a 1 : 2 molar ratio using the In‐Fusion cloning kit.

**Table 1:** (A) Primers used to produce the luciferase reporter constructs. Primers for In‐Fusion cloning have the tail sequences underlined, and bold text denotes the sequence of the restriction sites used for fusing the insert and vector. (B) Primers used for real‐time PCR in the analysis of chromatin immune‐precipitated DNA from LS174T wild‐type nuclear extract and in the analysis of mRNA from intestinal cell lines and primary colonic epithelial cells.

Primer name	Primer sequence	Restriction site
(A)		
Primers used for reporter constructs		
*IL33* promoter FW	GCCCCTTTGTTTCAAGGTCAC	
*IL33* promoter RW	TCATGGAGGGGGCAGAATTTAC	
Infusion *IL33* promoter FW	CTCGGCGGCC **AAGCTT** GCCCCTTTGTTTCAAGGTCACC	HindIII
Infusion *IL33* promoter RW	CCGGATTGCC **AAGCTT** TCATGGAGGGGGCAGAATTTAC	HindIII
*IL33* enhancer FW	ATGGTGGGGACTGGCATAAC	
*IL33* enhancer RW	AAAGAAAGATCTGTTAAGGGCCA	
Infusion *IL33* enhancer FW	ATAAGGATCC **GTCGAC** ATGGTGGGGACTGGCATAAC	Sal1
Infusion *IL33* enhancer RW	AAGGGCATCG **GTCGAC** AAAGAAAGATCTGTTAAGGGCC	SalI
(B)		
Primers used for ChIP analysis		
*IL33* ChIP FW	CAACCAGGAAATCACAGCAG	
*IL33* ChIP RW	GAGGCAATTTAGGGATGCAA	
Primers used for qPCR		
*IL33*FW	GTGGAAGAACACAGCAAGCA	
*IL33* RW	AAGGCAAAGCACTCCACAGT	
*RPLP0* FW	GCTTCCTGGAGGGTGTCC	
*RPLP0* RW	GGACTCGTTTGTACCCGTTG	

### Transfection with reporter constructs

LS714T and CDX2‐inducible LS174T cells were seeded in 24‐well plates at 100 000 cells/well and transfected after 24 h. For each well, a transfection mixture of 50 µL DNA/PEI mix was prepared, consisting of 2 µm polyethyleneimine (PEI) (Alfa Aesar, Heysham, UK) diluted in 150 mm NaCl to 25 µL and 300 ng plasmid DNA diluted with 150 mm NaCl to 25 µL. The transfection mix contained 50 ng CMV promoter‐driven expression plasmid for either the *IL33* promoter or both the promoter and enhancer. Fifty nanogram of an empty pGL4.10 CMV expression plasmid was used as a no‐insert control. Twenty‐five nanogram CMV‐LacZ plasmid was added to the mix as an internal control of transfection efficiency, and 225 ng pBluescript SK+ II was used as inactive DNA to reach 300 ng DNA/well. Volume was adjusted with 150 mm NaCl, and the transfection mix was incubated for 60 min at room temperature. The transfection mix was added in drops to the wells, followed by light shaking. After the transfection, the plates were centrifuged at 390 ***g*** for 5 min and incubated overnight at 37 °C and 5% CO_2_. The media were changed after 24 h, and cells were grown for an additional 24 h before assayed for luciferase and β‐galactosidase activity.

### Reporter construct activity

The cells were rinsed three times with 1× PBS and lysed with 130µL TROPIX lysis solution (Thermo Fisher Scientific) added 0.5 mm DTT by incubation for 10 min on ice. Ten microlitre of lysate was transferred to a 96‐well light plate, whereafter luciferase activity and β‐galactosidase activity were measured using 5‐s integration time and 2‐s delay using the Dual‐Light™ Luciferase & β‐Galactosidase Reporter Gene Assay System (Thermo Fisher Scientific) in GloMax luminometer.

### Chromatin immunoprecipitation assays

LS174T wild‐type cells were cultured until 5 days postconfluence in a 30 × 30 cm culture dish and were cross‐linked and sonicated as described previously to generate fragments of ~ 0.2 to 1.2 kb [11]. Briefly, four replicate immunoprecipitations (IP) were performed overnight at 4 °C with an antibody against human CDX2 (α‐CDX2 clone CDX2‐88; #MU392A‐UC, BioGenex, Fremont, CA, USA) or, as a negative control, an antibody against influenza hemagglutinin (HA) (rabbit polyclonal α‐HA, Y‐11 X, #SC‐805 X; Santa Cruz Biotechnology Inc, Heidelberg, Germany). Immune complexes were recovered with 50 µL protein A/G beads (Invitrogen, Grand Island, NY, USA). Real‐time PCR was carried out using DNA immunoprecipitated with CDX2 and HA or on non‐IP material from LS174T wild‐type cells (Input; representing 1% of the total amount used in IP) using primers designed for the peak in the *IL33* region (Table 1B).

### Protein extraction and immunoblotting

Wild‐type and CDX2‐inducible LS174T cells were seeded in 6‐well plates at 300 000 cells/well. After 48 h, the media were changed to media with varying concentrations of doxycycline. At 72 h, cells were rinsed with cold PBS and lysed in RP1 lysis buffer (Macherey‐Nagel, Düren, Germany) and proteins were purified. Protein concentrations were determined by the Bradford analysis (Bio‐Rad, Hercules, CA, USA). 20 µg protein was mixed 1 : 4 (v/v) with Bolt loading buffer and 1 : 10 (v/v) with Bolt sample reducing agent (Thermo Fisher Scientific). Samples were incubated at 95 °C for 10 min and loaded on a Bolt 4–12% Bis‐Tris Plus SDS gel, and electrophoresis was performed in 1× Bolt MOPS running buffer (Thermo Fisher Scientific) for 90 min at 100 V. Gels were transferred by wet electro‐transfer to PVDF membranes for 60 min at 25 V and max 100 mA in 1× NuPAGE transfer buffer (Thermo Fisher Scientific). Membranes were blocked with 5% dry skim milk in wash buffer (1× PBS with 0.1% Tween‐20) for 1 h at room temperature, washed with wash buffer 3 × 7 min, and incubated ON at 4 °C with primary antibody diluted in dilution buffer (2.5% skim milk in wash buffer). Membranes were washed 3 × 7 min and incubated with secondary antibodies for 1 h at room temperature and washed 3 × 7 min. Bands were visualized with SuperSignal™ West Dura Extended Duration Substrate (Thermo Fisher Scientific). Primary antibodies were rabbit anti‐IL‐33 (1 : 1000; #ab207737; Abcam, Cambridge, UK), mouse monoclonal anti‐CDX2 (1 : 1000; #MU392A‐UC; BioGenex), rabbit anti‐vinculin (1 : 5000; #ab129002; Abcam), and mouse anti‐glyceraldehyde 3‐phosphate dehydrogenase (GAPDH) (1 : 20 000; #10R‐G109a; Fitzgerald, Acton, MA, USA). The horseradish peroxidase‐labeled secondary antibodies were either goat anti‐mouse (1 : 10 000; #32230) or goat anti‐rabbit antibodies (1 : 10 000; #32260) (Thermo Fisher Scientific).

### Statistical analysis

Analyses were carried out using a two‐tailed Student's *t*‐test or one‐way ANOVA with multiple comparisons using Dunnett's test or Tukey's test. *P*‐values < 0.05 were considered significant. Values are presented as means with SD.

## Results and Discussion

**Fig. 1:**
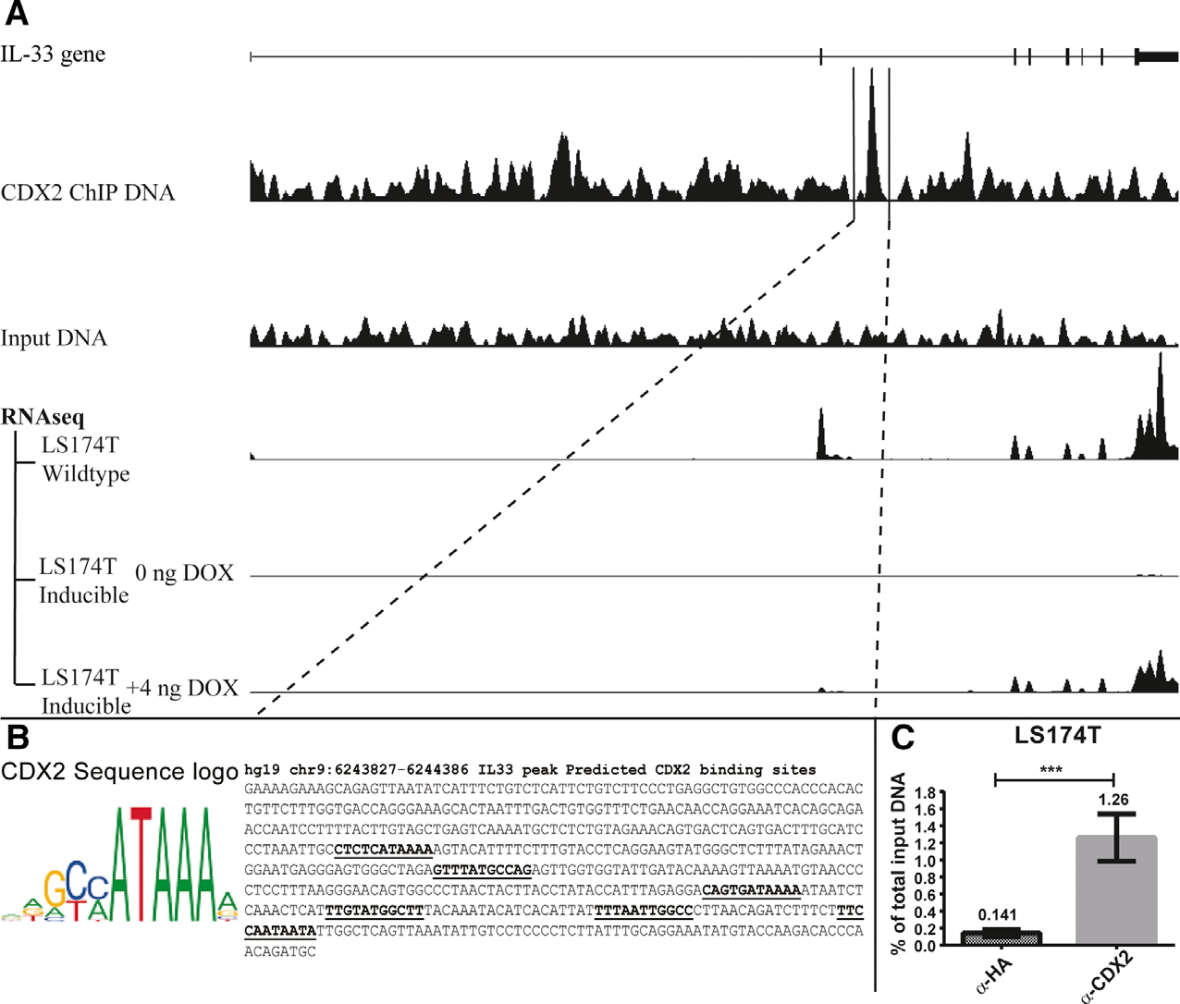
*IL33* is a potential CDX2 target in LS174T cells. (A) View of the 42 198 bp *IL33* gene (uc003zjt.3, NM_033439) from the UCSC Genome Browser assembly Feb. 2009 GRCh37/hg19. Imported data tracks include CDX2 ChIP‐seq reads of both immunoprecipitated and input DNA (representing 1% of the total amount used in IP) signal, shown as density graphs [9]. RNA‐seq data from deep sequencing transcriptomic analysis from wild‐type and CDX2‐inducible LS174T cells are shown with or without induction with 4 ng·mL^−1^ doxycycline for 24 h [9]. Data are displayed as density graphs. (B) The position weight matrix of the CDX2‐binding motif [ID: MA0465.1] from the JASPAR database (http://jaspar.genereg.net/) [13] including six (bold and underlined) predicted CDX2 target sites in the sequence of the major CDX2 binding element at the marked area (chr9:6243827–6244384) in the *IL33* gene. (C) qPCR of CDX2‐ and HA (negative control)‐immunoprecipitated chromatin from LS174T wild‐type cells using primer pairs located within the CDX2 ChIP‐seq peak in the *IL33* locus. The relative enrichment of CDX2 binding is shown as a percentage of total input DNA and is represented as means with SD error bars (*n* = 4), ***P < 0.001 using two‐tailed unpaired Student's *t*‐test.

We investigated whether the intestinal transcription factor CDX2 regulates *IL33* gene expression in intestinal epithelial cells. Initially, we analyzed previously published [9] genome‐wide chromatin‐immunoprecipitated sequence data (ChIP‐seq) from the LS174T cell line for the relative abundance of CDX2 in the *IL33* gene. We discovered a potential enhancer region in intron 2 with a clear CDX2 peak and predicted CDX2 binding sites (Fig. 1). Sequence analysis identified six potential CDX2‐binding sites within the *IL33* ChIP‐seq peak, indicating a potential regulatory region (Fig. 1B). After validating that CDX2 binds in the potential enhancer using quantitative PCR of CDX2‐precipitated chromatin from LS174T cells (Fig. 1C), we investigated to which extent CDX2 regulates *IL33* expression in LS174T cells. Using RNA‐seq data from the same study, we compared the *IL33* mRNA expression in LS174T wild‐type and a CDX2‐inducible LS174T clone that only expresses CDX2 in response to doxycycline stimulation [9]. The RNA‐seq data revealed that IL‐33 expression was relatively high in wild‐type cells, almost abolished in CDX2 knockout cells, and partially recovered in cells where CDX2 was induced back to the endogenous level (Fig. 1A).

**Fig. 2:**
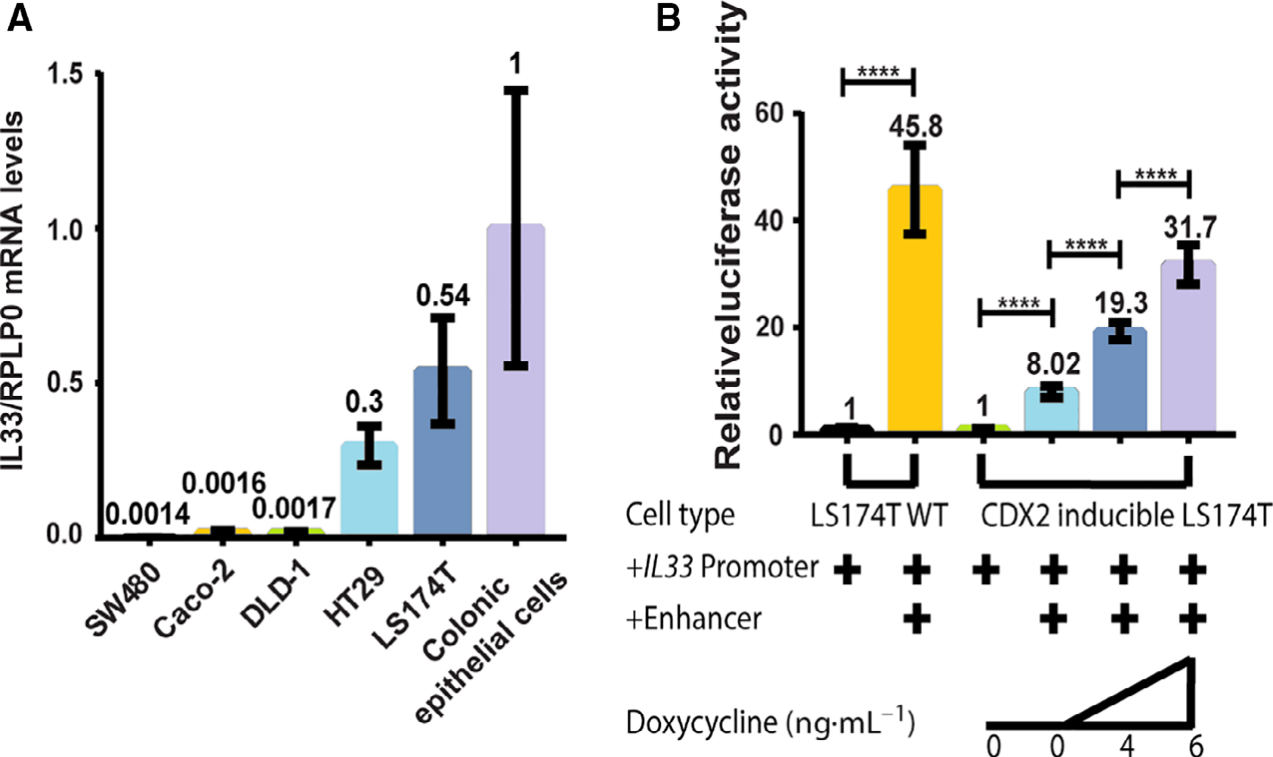
(A) Expression of *IL33* mRNA in intestinal cell lines and primary human colonic epithelial cells. Total RNA from SW480, Caco‐2, DLD‐1, HT29, LS174T cells, and colonic epithelial cells isolated from healthy humans. IL33 mRNA levels were measured by qRT‐PCR. Data were normalized to the reference gene RPLP0 and represented as mean values with SD error bars (*n* = 4) using one‐way ANOVA with multiple comparisons. (B) The relative luciferase activity of the *IL33* promoter and enhancer in LS174T wild‐type and CDX2‐inducible cells. + indicates that either the *IL33* promoter or enhancer sequence was inserted into the luciferase construct sequence. The final concentration of doxycycline present in the growth media is shown below. The activity was normalized to the constructs with the *IL33* promoter. Bars represent mean values with SD error bars. ****P < 0.0001, *n* = 4 using one‐way ANOVA with multiple comparisons.

We then measured the expression of *IL33* transcripts by qRT‐PCR in several well‐characterized intestinal epithelial cell lines and in primary colonic epithelial cells isolated from healthy subjects. To characterize the epithelial‐specific expression of the primary cells, we used an isolation method that minimizes fibroblast contamination [10]. The highest expression level of *IL33* was found in primary colonic epithelial cells, closely followed by LS174T and HT29 cells, while the *IL33* expression was either undetected or very low in SW480, DLD‐1, and Caco‐2 cell lines (Fig. 2A).

To further investigate the regulatory effect of CDX2 on *IL33* expression, we analyzed the IL‐33 promoter activity by constructing luciferase reporter plasmids containing the *IL33* promoter with or without the potential enhancer region from intron 2 and transfected them into LS174T wild‐type and the CDX2‐inducible LS174T clone. In the wild‐type cells, the potential enhancer region increased the *IL33* promoter activity more than 45‐fold from the promoter level (Fig. 2B), which demonstrates that it is a powerful enhancer of *IL33* in LS174T cells. In the unstimulated CDX2‐inducible LS174T cells expressing no CDX2, the relative enhancer only increased reporter gene expression by eightfold. This indicates that the enhancer activity, to a certain degree, is regulated by other factors than CDX2. However, when CDX2 was induced with 4 or 6 ng·mL^−1^ doxycycline for 24 h, the effect of the enhancer increased by 19‐fold and 32‐fold, respectively, demonstrating a CDX2‐dependent upregulation of the *IL33* promoter activity.

**Fig. 3:**
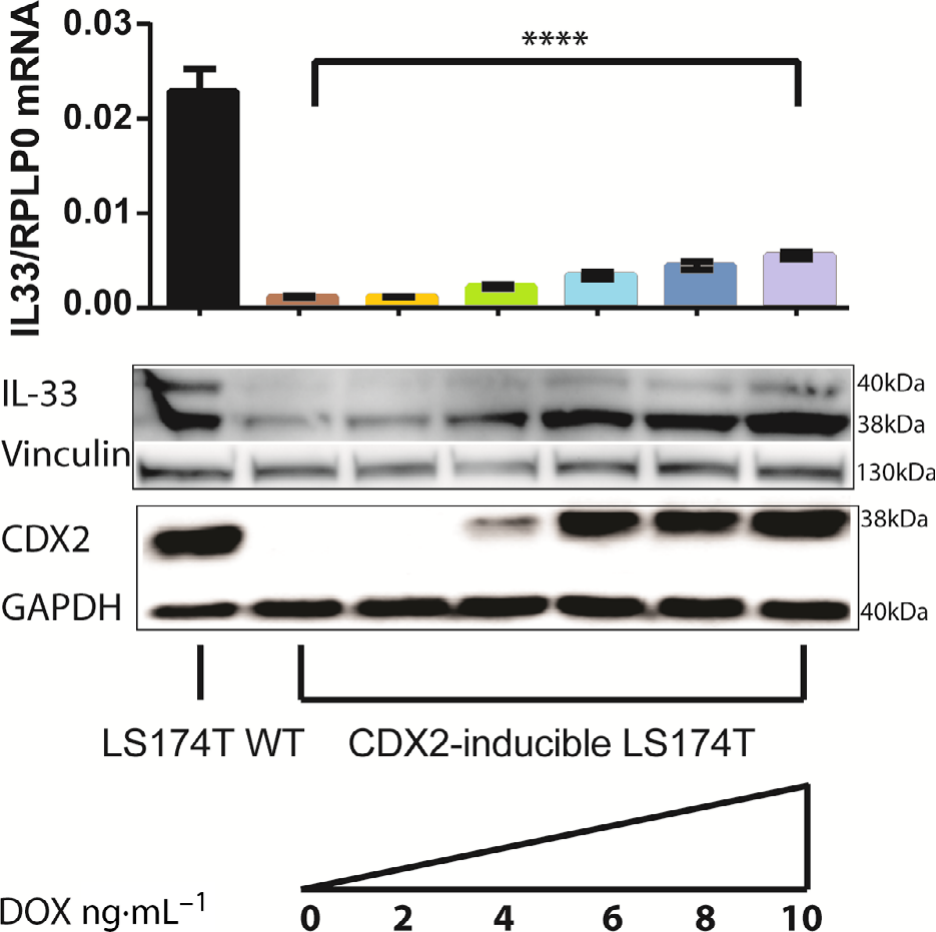
(Top) Quantitative RT‐PCR of *IL33* mRNA expression in LS174T wild‐type and CDX2‐inducible LS174T cells exposed to increasing concentrations of doxycycline, normalized to the reference gene RPLP0. CDX2 expression was induced by incubation with doxycycline for 24 h. Data are represented as mean values with SD error bars, (*n* = 4), ****P < 0.0001 using one‐way ANOVA with multiple comparisons. (Bottom) Western blot of the CDX2 and IL‐33 protein level in LS174T wild‐type and CDX2‐inducible LS174T cells after exposure to different concentrations of doxycycline for 24 h. GAPDH and vinculin were used as loading controls.

Subsequently, we treated CDX2‐inducible LS174T cells with increasing concentrations of doxycycline and measured the *IL33* mRNA expression using quantitative RT‐PCR and IL‐33 and CDX2 protein expression using western blotting analysis (Fig. 3). Similar to the RNA‐seq, we observed a relatively high *IL33* mRNA and protein expression in LS174T wild‐type cells and much lower expression in CDX2 knockout cells. Furthermore, we observed a doxycycline concentration‐dependent increase in *IL33* mRNA and protein expression. Interestingly, another study has found a significantly reduced *IL‐33* mRNA expression (GEO: GSE24633) in the intestines of mice where CDX2 was conditionally knocked out compared with CDX2‐positive control mice [12].

To determine whether IL‐33 protein was excreted from the cells during CDX2 induction, we analyzed supernatants from LS174T cells induced with 0–14 ng·mL^−1^ doxycycline for 24 h using Luminex analysis but found no detectable level of IL‐33 protein in the media (data not shown). Thus, our data suggest that the IL‐33 protein is retained intracellularly in the intestinal epithelial cells under unstimulated conditions.

This study provides the first evidence that the intestinal‐specific transcription factor CDX2 regulates the expression of *IL33*. Furthermore, we identified an intronic enhancer of *IL33* gene expression in LS174T cells. We suggest that CDX2 adds an intestinal‐specific layer of transcriptional control to the expression of IL‐33, potentially placing CDX2 as a key factor controlling the intestinal expression of an important cytokine with ‘alarmin’ function. Further validation from in vivo studies in mice or human organoid systems could strengthen this conclusion.

## Conflict of interest

The authors declare no conflict of interest.

## Author contributions

SL designed the study, performed experiments, analyzed data, obtained funding, and drafted the paper. JBS obtained patient samples, ran experiments, and drafted the paper. JD performed data analysis and drafted the paper. KD performed data analysis and drafted the paper. CHN performed Luminex assays, performed data analysis and drafted the paper. EPB cocreated the inducible cell line, performed data analysis, and drafted the paper. OBP performed data analysis and drafted the paper. MC designed the study, performed experiments, analyzed data, obtained funding, and drafted the paper obtained patient samples. JTT designed the study, obtained funding, performed data analysis, and drafted the paper. All authors discussed the results and contributed to the final manuscript.

## Data Availability

The RNA‐seq and ChIP‐seq data shown from Pinto *et al*. are uploaded at the NIH GEO server: http://www.ncbi.nlm.nih.gov/geo/ and are accessible using the following accession number GSE97273.
